# Indigo Carmine Hemodynamic Studies to Treat Vasoplegia Induced by Compound 48/80 in a Swine Model of Anaphylaxis

**DOI:** 10.21470/1678-9741-2020-0622

**Published:** 2022

**Authors:** Agnes Afrodite S Albuquerque, Andrea Carla Celotto, Christiane Becari, Marelaine Prandi, Jessyca M Barbosa, Francisco Moreira Neto, Maria Cecília Jordani, Paulo Roberto B Evora

**Affiliations:** 1 Department of Surgery and Anatomy, Faculdade de Medicina de Ribeirão Preto, Universidade de São Paulo, Ribeirão Preto, São Paulo, Brazil.; 2 Faculdade de Ciências da Saúde de Barretos Dr. Paulo Prata, Barretos, São Paulo, Brazil.

**Keywords:** Indigo Carmine, Anaphylaxis, Anaphylactic Shock, Vasoplegia, Nitric Oxide, Methylene Blue

## Abstract

**Introduction:**

There are many reasons to believe that the nitric oxide/guanosine 3’5’ - cyclic monophosphate (or NO/cGMP) pathway on vasoplegic states is underestimated. To study indigo carmine (IC) as an alternative to methylene blue was the investigation rationale.

**Methods:**

The IC (3mg/kg intravenous infusion) study protocol included five experimental groups; 1) Control group — saline was injected at 0 and 10 minutes; 2) IC group — IC was injected at 0 and saline at 10 minutes; 3) compound 48/80 (C48/80) group — C48/80 was injected at 0 minute and saline at 10 minutes; 4) C48/80 + IC group — C48/80 was injected at 0 minute and IC at 10 minutes; and 5) IC + C48/80 group — IC was injected at 0 minute and C48/80 at 10 minutes. The studies were carried out by registering and measuring hemodynamic and blood gasometric parameters, including continuous cardiac output.

**Results:**

1) The effects of the drugs (IC and C48/80) were more evident in the first 20 minutes of recording; 2) hypotensive responses were more pronounced in the C48/80 groups; 3) IC isolated or applied before C48/80 caused transient pulmonary hypertension; and 4) after the first 20 minutes, the pressure responses showed stability with apparent hypotension more pronounced in the C48/80 groups. Clinical observations showed significant hemodynamic instability and catastrophic anaphylactic reactions (agitation, pulmonary hypertension, severe bronchospasm, urticaria, high-intensity cyanosis, violent gastric hypersecretion, and ascites).

**Conclusion:**

A global results analysis showed differences between groups only in the first 20 minutes of the experiments.

**Table:** 

Abbreviations, acronyms & symbols	
ACh	= Acetylcholine
C	= Control
C48/80	= Compound 48/80
cGMP	= Guanosine 3’5’ - cyclic monophosphate
CO	= Cardiac output
COI	= Cardiac output index
CVP	= Central venous pressure
Hb	= Hemoglobin
Ht	= Hematocrit
IC	= Indigo carmine
MAP	= Mean arterial pressure
MB	= Methylene blue
MCVP	= Mean central venous pressure
MPAP	= Mean pulmonary arterial pressure
NO	= Nitric oxide
NOx	= Nitrite/nitrate
pCO_2_	= Partial carbon dioxide
PCP	= Pulmonary capillary pressure
pO_2_	= Partial pressure of oxygen
PVR	= Pulmonary vascular resistance
PVRI	= Pulmonary vascular resistance index
S	= Saline
SO_2_	= Oxygen saturation
SNP	= Sodium nitroprusside
SVR	= Systemic vascular resistance
SVRI	= Systemic vascular resistance index
USA	= United States of America

## INTRODUCTION

Recently, we have noticed in the medical literature the case report of a patient undergoing methylene blue (MB) infusion to check for ureteral perviousness. MB infusion caused a transient and moderate increase in systemic vascular resistance (SVR) and mean arterial pressure (MAP). In a different way, a more significant and longer-lasting improvement in these parameters with indigo carmine (IC) infusion was observed^[1]^. We also read the first reported case of IC use to treat vasoplegic syndrome in cardiac surgery — in a 78-year-old patient under cardiopulmonary bypass for myocardial revascularization. IC was successfully used after fluid overload, norepinephrine, vasopressin, and MB failed to control arterial hypotension^[2]^. These two observations suggest that IC may be an alternative agent for reversing vasodilation in conditions such as anaphylaxis and septic shock. However, IC investigations as a therapeutic option or experimental tool are scarce or inexistent.

In addition, IC should be a great tool to study *in vitro* vascular reactivity. Briefly, 1) IC inhibits endothelium-dependent vasorelaxation induced by acetylcholine (ACh), histamine, and calcium ionophore A23187 and endothelium-independent vasorelaxation caused by sodium nitroprusside (SNP) in rat aortic rings; 2) inhibition is selective for agents that produce vasorelaxation in association with guanosine 3’5’ - cyclic monophosphate (cGMP) release; 3) cyclooxygenase activity does not appear to contribute; and 4) the site of inhibitory effect on endothelial NO production is probably distal to membrane receptors and involves cytosolic calcium availability^[3]^.

These IC effects may be useful as an attractive experimental tool and perhaps help save lives. Therefore, this study aimed to test the systemic and pulmonary hemodynamic properties of IC experimentally.

## METHODS

The animal procedures and experimental protocols of this study were approved by the Ethics Committee on Animal Experimentation (CETEA) of the Faculdade de Medicina de Ribeirão Preto (or FMRP) (23/2015).

### Animals

Male Dalland pigs (22-26 kg) were induced to anesthesia with an intramuscular injection of xylazine (10 mg/kg) associated with ketamine (50 mg/kg) in the quadriceps muscle of either the hind paws. The anesthesia was maintained by intravenous (right dorsal vein) reapplications of 1/3 of the initial dose every 30 minutes. Volemia maintenance was achieved with intravenous infusion of sodium chloride 0.9% (5 ml/kg/h).

### Experimental Design

The study protocol included five experimental groups. IC dosage was 3 mg/kg (intravenous infusion).

1. Control (C) group — saline (S) was injected at 0 and 10 minutes.
2. IC group — IC was injected at 0 and S at 10 minutes.
3. Compound 48/80 (C48/80) group — C48/80 was injected at 0-minute S at 10 minutes.
4. C48/80 + IC group — C48/80 was injected at 0 minute and IC at 10 minutes.
5. IC + C48/80 group — IC was injected at 0 minute and C48/80 at 10 minutes.

### Hemodynamic Parameters

A Swan-Ganz CCOmbo CCO/SvO_2_ 744HF75 (Edwards Lifesciences, California, United States of America [USA]) catheter was placed in the right jugular vein and into the lumen of the main pulmonary artery. The left carotid artery was simultaneously catheterized. MAP, pulmonary arterial pressure (PAP), pulmonary capillary pressure (PCP), and central venous pressure (CVP) were recorded by the MP System 100 A (BioPac System, Inc., California, USA). Cardiac output (CO), SVR, and pulmonary vascular resistance (PVR) were obtained by the Vigilance System (Edwards Lifesciences LLC, California, USA). After instrumentation, a period of 20 minutes was allowed for anesthesia stabilization when hemodynamic parameters and clinical conditions were continuously recorded.

### Nitrite/nitrate (NOx)

Plasma NOx dosage was carried out using chemiluminescence concentrations (Analyzer 280i NOA [Sievers, Boulder, Colorado, USA]).

### Gasometric, Lactate, and Electrolytic Analysis

Arterial blood samples were collected from each animal’s carotid artery. Biochemical measurements of pH, partial pressure of carbon dioxide (or pCO_2_), partial pressure of oxygen (pO_2_) and plasma concentration of bicarbonate ion (or HCO_3_-) were performed by a previously calibrated Gem Premier 3000 (Instrumentation Laboratory Co., Bedford, Massachusetts, USA) using iQM 150 GEM Premier (iQM Instrumentation Laboratory Co., Bedford, Massachusetts, USA). The values of hemoglobin (Hb), hematocrit (Ht), oxygen saturation (SO_2_), lactate, and electrolytes were measured using the same arterial blood sample.

### Statistical Analysis

Results were expressed as means ± standard error of mean and analyzed by two-way analysis of variance (ANOVA), and, when necessary, Dunnett post-test, using Prism 8.0 (GraphPad Software Incorporated, 1999). Values were considered to be statistically significant when *P*-value < 0.05.

## RESULTS

### Mean Arterial Pressure (MAP)

**Fig. 1:**
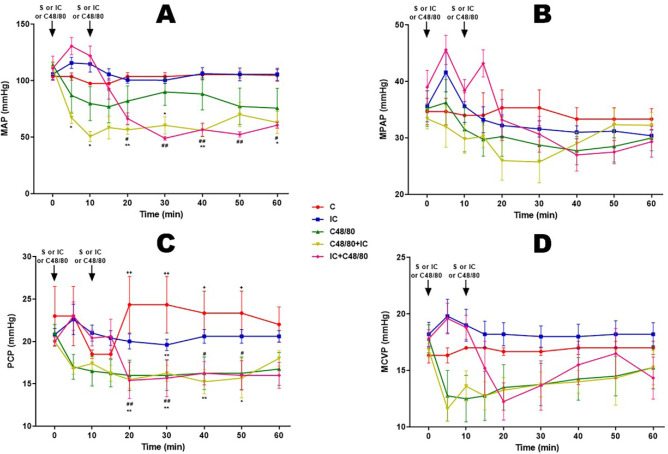
Blood pressures. Effect of anaphylactic shock caused by compound 48/80 (C48/80) and treated with indigo carmine (IC) in pigs. A) Mean arterial pressure (MAP); B) mean pulmonary arterial pressure (MPAP); C) pulmonary capillary pressure (PCP); D) mean central venous pressure (MCVP). At minute 0, the control (C) group received saline (S), the C48/80 and C48/80 + IC groups received C48/80, the IC and IC + C48/80 groups received IC. At minute 10, the C, IC, and C48/80 groups received S, the C48/80 + IC group received IC, and the IC + C48/80 group received C48/80. Data represent means ± standard error of mean and analyzed by two-way analysis of variance (ANOVA), Dunnett post-test (n=5).^*/+/#^P<0,05;^**/++/##^P<0,01 (*C vs. C48/80 + IC; + C vs. C48/80;^#^C vs. IC + C48/80).

MAPs remained stable in groups C and IC (around 100 mmHg). Early arterial hypotension occurred in two groups (C48/80, C48/80 + IC); and in group IC + C48/80, the MAP drop started after the first 10 minutes, when the second injection was performed. Arterial hypotension was more pronounced in groups receiving C48/80 in association with IC (Figure 1A).

### Mean Pulmonary Arterial Pressure (MPAP)

MPAP remained stable only in group C (around 35 mmHg). And after initial pulmonary hypertension, group IC showed similar evolution to group C. In group C48/80 + IC, there was an immediate and marked fall in MPAP, starting spontaneous recovery after 30 minutes. whereas after initial hypotension, group C48/80 showed similar behavior to group IC and group IC + C48/80 maintained pulmonary hypertension for the first 15 minutes when spontaneous fall began (Figure 1B).

### Mean Pulmonary Capillary Arterial Pressure (PCP)

PCP remained stable in group IC, and group C showed significantly higher values. The groups that received C48/80 or C48/80 + IC had decreased pressure since the beginning of registration; and the IC + C48/80 group after the 20^th^ minute, it remained stable (Figure 1C).

### Central Venous Pressure (MCVP)

MCVP in the first 10 minutes remained relatively stable in groups C, IC, and IC + C48/80. C48/80 and IC + C48/80 groups showed an immediate decrease in CVP and the IC + C48/80 group maintained the CVP for five minutes when it started to fall (Figure 1D).

### Cardiac Output (CO)

**Fig. 2:**
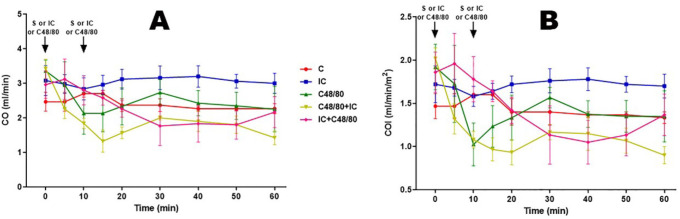
Cardiac output. Effect of anaphylactic shock caused by compound 48/80 (C48/80) and treated with indigo carmine (IC) in pigs. A) Cardiac output (CO); B) cardiac output index (COI). At minute 0, the control (C) group received saline (S), the C48/80 and C48/80 + IC groups received C48/80, the IC and IC + C48/80 groups received IC. At minute 10, the C, IC, and C48/80 groups received S, the C48/80 + IC group received IC, and the IC + C48/80 group received C48/80. Data represent means ± standard error of mean and analyzed by two-way analysis of variance (ANOVA), Dunnett post-test (n=5). No statistical difference was observed.

Both CO and cardiac output index (COI) showed similar results. In groups C and IC, it was stable until the end. After the injection of C48/80, in the C48/80 and C48/80 + IC groups, CO and COI decreased until the 10^th^ minute and then stabilized. And IC injection before C48/80 in IC + C48/80 group did not improve either CO or COI (Figures 2A and 2B).

### Resistances

**Fig. 3:**
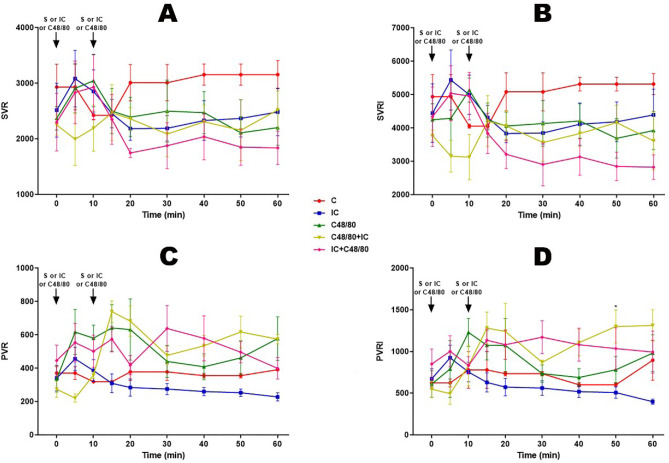
Vascular resistances. Effect of anaphylactic shock caused by compound 48/80 (C48/80) and treated with indigo carmine (IC) in pigs. A) Systemic vascular resistance (SVR); B) systemic vascular resistance index (SVRI); C) pulmonary vascular resistance (PVR); D) pulmonary vascular resistance index (PVRI). At minute 0, the control (C) group received saline (S), the C48/80 and C48/80+IC groups received C48/80, the IC and IC + C48/80 groups received IC. At minute 10, the C, IC, and C48/80 groups received S, the C48/80 + IC group received IC, and the IC + C48/80 group received C48/80. Data represent means ± standard error of mean and analyzed by two-way analysis of variance (ANOVA), Dunnett post-test (n=5). *P<0,05; (*C vs. C48/80+IC).

Almost all groups (C, IC, C48/80, IC + C48/80) showed a slight increase or stability in SVR and SVRI in the first 10 minutes while C48/80 + IC group showed a decrease in the same time; after 15 minutes the IC, C48/80, IC + C48/80, and C48/80 + IC groups remained stable while the C group increased and remained stable (Figures 3A and 3B). In C and IC groups, the PVR and PVRI were stable until the end, whereas in C48/80 and C48/80 + IC groups, there was an increase after 10 minutes, showing a decrease soon after and another increase after 30 minutes, tending to pulmonary hypertension, as opposed to the IC + C48/80 group revealing the fall (Figures 3C and 3D).

### Acid-Base Balance

**Fig. 4:**
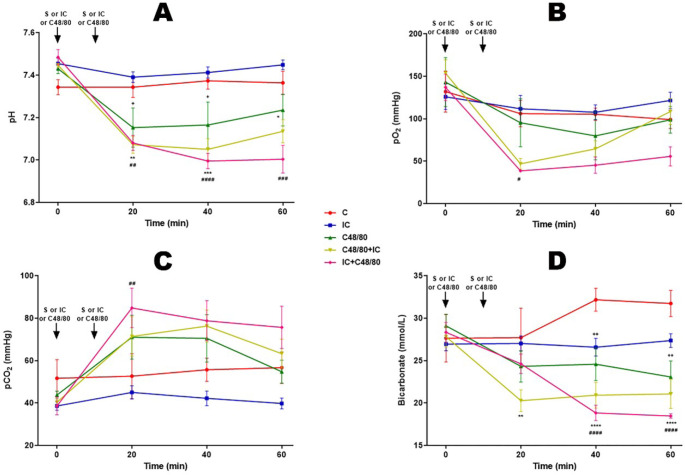
Acid-base data. Effect of anaphylactic shock caused by compound 48/80 (C48/80) and treated with indigo carmine (IC) in pigs. A) pH; B) partial pressure of oxygen (pO2); C) Partial pressure of carbon dioxide (pCO2); D) bicarbonate. At minute 0, the control (C) group received saline (S), the C48/80 and C48/80 + IC groups received C48/80, the IC and IC + C48/80 groups received IC. At minute 10, the C, IC, and C48/80 groups received S, the C48/80 + IC group received IC, and the IC + C48/80 group received C48/80. Data represent means ± standard error of mean and analyzed by two-way analysis of variance (ANOVA), Dunnett post-test (n=5).^+/#^P<0,05;^**/++/##^P<0,01;^***/###^P<0,001;^****/####^P<0,0001 (*C vs. C48/80 + IC; + C vs. C48/80;^#^C vs. IC + C48/80).

Groups receiving C48/80, C48/80 + IC, and IC + C48/80 showed a tendency to acidosis (Figures 4A and 4D), hypoxia (Figure 4B), and hypoventilation with respiratory acidosis (Figure 4C).

### Hemoglobin, Hematocrit, SO2, and Lactate

**Fig. 5:**
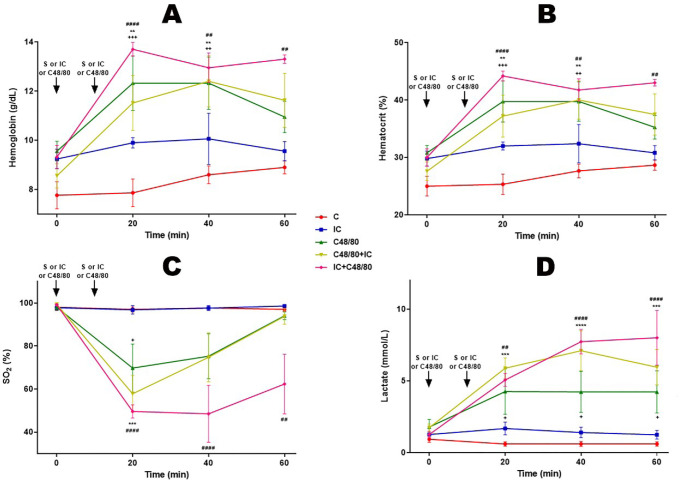
Hemoglobin, hematocrit, oxygen saturation and lactate. Effect of anaphylactic shock caused by compound 48/80 (C48/80) and treated with indigo carmine (IC) in pigs. A) Hemoglobin; B) Hematocrit; C) Oxygen saturation (SO2); D) Lactate. At minute 0, the control (C) group received saline (S), the C48/80 and C48/80+IC groups received C48/80, the IC and IC+C48/80 groups received IC. At minute 10, the C, IC and C48/80 groups received S, the C48/80+IC group received IC and the IC+C48/80 group received C48/80. Data represent means ± standard error of mean and analyzed by two-way analysis of variance (ANOVA), Dunnett post-test (n=5).^+^ P<0,05;^*/++/##^ P<0,01;^+++/*^ P<0,001;^**/####^ P<0,0001 (C vs. C48/80+IC; + C vs. C48/80;^#^C vs. IC+C48/80).

Groups receiving C48/80, C48/80 + IC, and IC + C48/80 had higher Hb and Ht values (Figures 5A and 5B). Since there was no significant blood loss, it is suggested that in these groups hemoconcentration occurred due to increased capillary permeability. In these groups, there was also a tendency to decrease SO_2_, compatible with a decrease in CO (Figure 5C), with an increase in lactate values (Figure 5D).

### Urea, creatinine, and nitrate

**Fig. 6:**
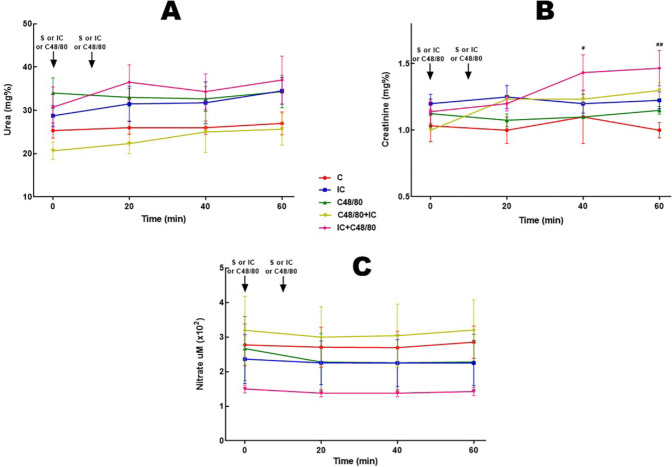
Urea, creatinine, and nitrate. Effect of anaphylactic shock caused by compound 48/80 (C48/80) and treated with indigo carmine (IC) in pigs. A) Urea; B) creatinine; C) nitrate. At minute 0, the control (C) group received saline (S), the C48/80 and C48/80 + IC groups received C48/80, the IC and IC + C48/80 groups received IC. At minute 10, the C, IC and C48/80 groups received S, the C48/80 + IC group received IC, and the IC + C48/80 group received C48/80. Data represent means ± standard error of mean and analyzed by two-way analysis of variance (ANOVA), Dunnett post-test (n=5).^#^P<0,05;^##^P<0,01. (^#^C vs. IC + C48/80).

The dosages of urea and nitrate did not present results with statistically significant differences. On the other hand, the creatinine dosage in the IC+C48/80 group showed a significant increase after 20 minutes (Figure 6A, 6B, and 6C).

### Electrolytes

**Fig. 7:**
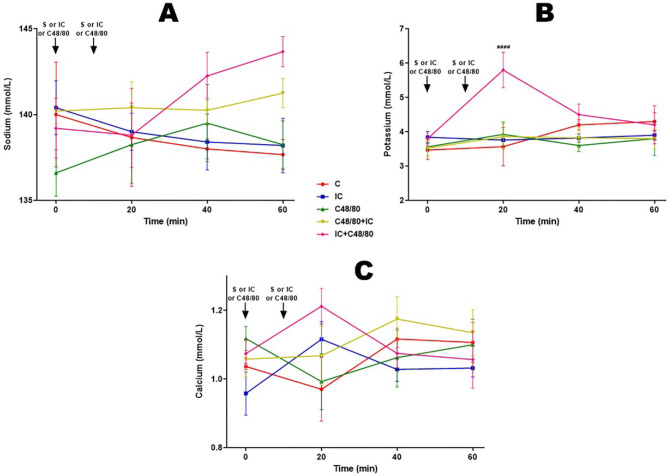
Electrolytes. Effect of anaphylactic shock caused by compound 48/80 (C48/80) and treated with indigo carmine (IC) in pigs. A) Sodium; B) potassium; C) calcium. At minute 0, the control (C) group received saline (S), the C48/80 and C48/80 + IC groups received C48/80, the IC and IC + C48/80 groups received IC. At minute 10, the C, IC, and C48/80 groups received S, the C48/80 + IC group received IC, and the IC + C48/80 group received C48/80. Data represent means ± standard error of mean and analyzed by two-way analysis of variance (ANOVA), Dunnett post-test (n=5).^####^P<0,0001. (^#^C vs. IC + C48/80).

Electrolytes’ dosages did not present particular tendencies regardless of protocol. There are statistically significant differences only at 20 minutes of potassium dosage in the IC + C48 / 80 group. Data are shown in Figure 7A, 7B, and 7C.

## DISCUSSION

In 1997, we reported an unprecedented experience with MB to treat anaphylactic shock from iodine contrast, after high doses of adrenaline and hydrocortisone failed^[4]^. After this observation we presented six cases of patients who were reversed, "in extremis"^[5]^, with clinical experience showing favorable data for MB use in anaphylaxis reversal.

Based on these anecdotal observations, we initiated experimental protocols to vary animal species and maintain C48/80 as an anaphylactic inducer. Our data showed that C48/80 was effective in inducing anaphylactic shock in pigs since both MAP and CO decreased after C48/80 administration. Curiously, in the first two minutes after the C48/80 injection, the animals presented a hypertensive crisis (without statistical significance) and a possible explanation for this is the direct stimulation of the hypothalamus-hypophysis axis. The CO reduction could be related to the negative inotropic effect of the C48/80^[8,9]^ and could explain the CVP trend to increase. As a line of research, we keep using this experimental model, including the present investigation, even knowing that most of the animals exposed to C48/80 presented cutaneous hyperemia, vomits, sphincters liberation, livedo reticularis, cyanosis, ascites, and intestinal ischemia and distention.

Previous research in our laboratory showed that MB reduced hypotension and increased survival time in a C48/80-induced anaphylactic shock model in rabbits^[6]^. Otherwise, intravenous infusion of MB alone in pigs caused no changes in the registered hemodynamic parameters but did not prevent or reverse the C48/80-induced anaphylactic shock in this model. Furthermore, as abovementioned, we verified that intravenous infusion of IC alone caused no changes in hemodynamic and clinical parameters showing that the administered MB dose (2-3 mg/kg) was safe in this experimental model. Our results corroborate other clinical data mentioning MB therapeutic safety in reversing catecholamine-resistant hypotension in systemic inflammatory response syndrome and anaphylaxis F^[4-7,9-14]^.

Based on this possibility, the present hemodynamic recording of systemic and pulmonary pressures was performed. However, the expected and severe pulmonary hypertension caused by IC injection renders its use in clinical practice unproven. IC injection causes immediate transient pulmonary hypertension. The primary IC data from the current survey, in a global analysis of all the studied data, showed only small differences between groups, without advantages when compared with our cumulated MB experience.

As time goes by, even American and European guidelines suggesting MB as a second choice for anaphylaxis treatment have been considered. Our long experience of saving lives with MB has led us to believe that the NO/cGMP pathway’s capitol role on vasoplegic states is underestimated. This feeling is enforced because MB, a drug over 100 years old, is the only drug option to be used clinically in humans. Therefore, to study IC as an alternative to MB was the rationale for the present investigation. However, the results were frustrating, as more IC efficiency was expected, due to its referred alpha-adrenergic stimulation. The main data from the current survey are quite similar with Menardi et al.^[15]^.

1. The effects of the drugs (IC and C48/80) were more evident in the first 20 minutes of recording the pressures.
2. Hypotensive responses were more pronounced in the groups that received C48/80.
3. IC isolated or applied before C48/80 caused transient pulmonary hypertension. This is a curious fact since the drugs applied separately showed only hypotension.
4. After the first 20 minutes, the pressure responses showed stability with apparent hypotension more pronounced in the groups that received C48/80.

IC did not produce severe disturbances in the basic acid balance, except for hypoventilation, which was promptly reversed by adjustment of breathing pain. In addition to its actions on NO/cGMP, IC is also an alpha agonist, which gives it an attractive property to treat vasoplegia induced by systemic inflammatory reactions. However, this vasoconstrictive property was not demonstrated. Therefore, based on the present pig model, IC cannot be considered as a routine option to treat vasoplegia associated with anaphylaxis. Perhaps when other treatment (epinephrine, corticoids, MB) fails, IC would be used as "rescue therapy".

During the experiment, clinical observations showed significant hemodynamic instability and catastrophic anaphylactic reactions (agitation, pulmonary hypertension, severe bronchospasm preventing animal ventilation, urticaria and high-intensity cyanosis, violent gastric hypersecretion, and a pig with massive ascites).

## CONCLUSION

As a conclusion, it is inferred that IC use is not safe, at least in pigs. Dye has been used primarily in the exploration of ureteral fistula in humans; it is possible to speculate that catastrophic observations may be related to the chosen experimental animal (pig). It is noteworthy that the changes in the parameters studied occurred in the groups that received C48/80, IC + C48/80, and C48/80 + IC.

Finally, we agree with Francuzik et al.^[14]^, which suggested the use of second-line medication, such as MB and vasopressin, in cases where two doses of adrenaline did not result in rapid normalization of anaphylaxis symptoms. Similarly, we suggest, in addition to many studies, that IC would be a rescue treatment for catastrophic anaphylactic reactions irresponsive to adrenaline and MB. Pharmacologically, IC would be more efficient than MB because it has an alfa-adrenergic effect. We have a word of caution, since the C48/80 associated with IC reactions in pigs was intense and dangerous. However, lifesaving use of IC has been reported^[12]^. The favorable data for IC use are no observations of clinical and experimental reactions to its infusions without association with C48/80.

**Table:** 

Authors’ Roles & Responsibilities	
AASA	Substantial contributions to the conception or design of the work; or the acquisition, analysis, or interpretation of data for the work; drafting the work or revising it critically for important intellectual content; agreement to be accountable for all aspects of the work in ensuring that questions related to the accuracy or integrity of any part of the work are appropriately investigated; final approval of the version to be published
ACC	Substantial contributions to the conception or design of the work; or the acquisition, analysis, or interpretation of data for the work; drafting the work or revising it critically for important intellectual content; final approval of the version to be published
CB	Substantial contributions to the conception or design of the work; or the acquisition, analysis, or interpretation of data for the work; drafting the work or revising it critically for important intellectual content; agreement to be accountable for all aspects of the work in ensuring that questions related to the accuracy or integrity of any part of the work are appropriately investigated; final approval of the version to be published
MP	Substantial contributions to the conception or design of the work; or the acquisition, analysis, or interpretation of data for the work; final approval of the version to be published
JMB	Substantial contributions to the conception or design of the work; or the acquisition, analysis, or interpretation of data for the work; final approval of the version to be published
FMN	Acquisition, analysis, or interpretation of data for the work; final approval of the version to be published
MCJ	Substantial contributions to the conception or design of the work; or the acquisition, analysis, or interpretation of data for the work; drafting the work or revising it critically for important intellectual content; final approval of the version to be published
PRBE	Substantial contributions to the conception or design of the work; or the acquisition, analysis, or interpretation of data for the work; drafting the work or revising it critically for important intellectual content; agreement to be accountable for all aspects of the work in ensuring that questions related to the accuracy or integrity of any part of the work are appropriately investigated; final approval of the version to be published
